# Modulation and Pharmacology of the Mitochondrial Permeability Transition: A Journey from F-ATP Synthase to ANT

**DOI:** 10.3390/molecules26216463

**Published:** 2021-10-26

**Authors:** Andrea Carrer, Claudio Laquatra, Ludovica Tommasin, Michela Carraro

**Affiliations:** Department of Biomedical Sciences, University of Padova, 35131 Padova, Italy; carrer.and@gmail.com (A.C.); claudiolaquatra@gmail.com (C.L.); ludovica.tommasin@studenti.unipd.it (L.T.)

**Keywords:** permeability transition, calcium, F-ATP synthase, adenine nucleotide translocator, cyclophilin D, mitochondrial channels

## Abstract

The permeability transition (PT) is an increased permeation of the inner mitochondrial membrane due to the opening of the PT pore (PTP), a Ca^2+^-activated high conductance channel involved in Ca^2+^ homeostasis and cell death. Alterations of the PTP have been associated with many pathological conditions and its targeting represents an incessant challenge in the field. Although the modulation of the PTP has been extensively explored, the lack of a clear picture of its molecular nature increases the degree of complexity for any target-based approach. Recent advances suggest the existence of at least two mitochondrial permeability pathways mediated by the F-ATP synthase and the ANT, although the exact molecular mechanism leading to channel formation remains elusive for both. A full comprehension of this to-pore conversion will help to assist in drug design and to develop pharmacological treatments for a fine-tuned PT regulation. Here, we will focus on regulatory mechanisms that impinge on the PTP and discuss the relevant literature of PTP targeting compounds with particular attention to F-ATP synthase and ANT.

## 1. Introduction

The permeability transition (PT) refers to an increased permeability of the inner mitochondrial membrane (IMM) to solutes in response to matrix Ca^2+^ which leads to matrix swelling. Although this process was initially considered as a direct consequence of membrane damages likely due to activation of phospholipases (PLAs) [1], the PT was then ascribed to the opening of a regulated channel, the so-called PT pore (PTP) [2,3,4]. The PTP is now defined as an unselective mitochondrial high conductance channel with an estimated diameter of 14 Å [5], allowing solutes of up 1.5 kDa to equilibrate across the membrane. Supporting evidence was provided by electrophysiological studies in mitoplasts which confirmed the existence of a Ca^2+^-activated high conductance channel and identified some peculiar characteristics, i.e., a maximal conductance of 1.3 nS, a variety of subconductance states, and the typical flickering activity (rapid oscillation between closed and open states) [6,7,8].

Although the molecular identity of the PTP is a long-standing mystery, some efforts have been made aiming to obtain a final picture of the pore components. An early model for PTP formation predicted a multiprotein complex including the core constituents adenine nucleotide translocator (ANT) and voltage-dependent anion channel (VDAC) orchestrated primarily by hexokinase 2 and cyclophilin D (CyPD), which would act as modulators [9]. However, gene inactivation studies led to the dismissal of this hypothesis, as reviewed in [10]. Recent advances provided new clues on the most promising candidates, indicating F-ATP synthase as a leading PTP component and re-evaluating the contribution of ANT in the PT process. In this review, we will focus on the role of F-ATP synthase and ANT in PTP formation, examine regulatory mechanisms, and discuss the relevant PTP pharmacology.

## 2. Modulation of the PTP

Over decades of studies, a plethora of endogenous modulators have been discovered to govern PTP opening, and while some regulatory sites have been identified, most of them remain largely unknown. Matrix Ca^2+^, also referred to as the “permissive” factor, results to be a strict requirement for PTP activation, although it might not always be sufficient to initiate the PT process. The minimum Ca^2+^ threshold necessary for PTP opening varies indeed among experimental conditions and depends on many additional factors that could change the sensitivity of the pore to the cation. For example, divalent cations other than Ca^2+^, such as Mg^2+^, Sr^2+^, and Ba^2+,^ delay PT occurrence, likely by competing with Ca^2+^ for the same binding site [11,12]. Adenine nucleotides that act synergistically with Mg^2+^ also contribute to PTP inhibition through a yet undefined mechanism. In early studies [2], Hunter and Haworth reported that the inhibitory effect of ADP on the PT is mediated by low- and high-affinity binding sites, the former being abolished by the ANT-specific ligand atractylate (ATR). Halestrap and co-workers proposed that the low-affinity binding site for ADP is located in the ANT as well [13]. In de-energized mitochondria, acidic (below 7) or alkaline (above 7.4) matrix pH mediates desensitization of the PTP, while in the first case, the inhibition occurs through the reversible protonation of matrix His residues [14], the basis of inhibition in the latter remains obscure. Of note, in energized mitochondria, an external acidic pH promotes rather than inhibits PTP opening by augmenting the uptake of inorganic phosphate (Pi) through the phosphate carrier [15]. Pi is one of the most puzzling PTP modulators and its mechanism of action has been only partially dissected. Despite the fact that Pi binds matrix Ca^2+^, sequestering it in the form of precipitates that decrease the free Ca^2+^ required for PTP opening [16,17], it readily induces pore opening. It has been proposed that Pi exerts its positive action when present in the form of polyphosphate aggregates, the loss of which significantly delays PT occurrence [18]. On the other hand, the PTP-inducing effect of Pi is only seen in the presence of CyPD, a mitochondrial peptidyl-prolyl cis-trans isomerase and one of the best-characterized PTP activators, as we will discuss in the following section. The ablation of CyPD in mammals indeed prevents PT activation by Pi and rather unmasks an inhibitory effect, which may depend on the lowered matrix-free Ca^2+^ concentration [19]. This effect of Pi was also documented for other species, i.e., *D. melanogaster* [20] and *S. cerevisiae* [21,22,23], in which mitochondrial CyPs do not appear to participate in PTP modulation.

The PTP is a voltage-dependent channel and promptly responds to membrane depolarization, which boosts its open probability [24], apparently mediated by voltage sensing residues that have not been fully characterized yet. Critical arginines are thought to tune a putative voltage sensor of the pore [25,26]. These residues appear not to reside in the ANT given that ANT-deficient mitochondria do undergo mitochondrial swelling upon carbonyl cyanide 4-(trifluoromethoxy)phenylhydrazone (FCCP) treatment [27] and that the arginine-specific reagent phenylglyoxal (PGO) does not affect ADP/ATP exchange properties [25]. However, permeabilized C2C12 myotubes devoid of ANT1 exhibited higher voltage-thresholds of PTP opening, suggesting that ANT might be involved in the voltage-sensing mechanism [28]. Among pore activators, fatty acids (FAs) deserve mention. FAs have been shown to be potent uncouplers of oxidative phosphorylation [29]. Whether FAs activate PTP opening through membrane depolarization or direct binding to PTP constituents is still not known, and we will address this aspect together with the effect of phospholipase inhibitors in an upcoming section. Thiol reagents that are also known positive regulators of PTP opening might act by shifting the gating potential of the pore to higher values, i.e., making pore opening more sensitive to small depolarizations [30]. Distinct active thiols have been identified by a careful phenotypic study using specific reagents. At least three sites contribute to control PTP opening by oxidation: two thiols exposed to the matrix side are apparently in equilibrium with the pyridine nucleotide and glutathione pools, are protected by micromolar concentrations of N-ethylmaleimide (NEM) and react with phenylarsine oxide (PAO), diamide (DIA) and *tert*-butyl hydroperoxide (TBH) [31]; a third site instead faces the intermembrane space (IMS) and reacts with copper-*o*-phenanthroline or with millimolar concentrations of NEM [32].

Although the modulation of the PTP has been extensively described, the lack of a detailed picture of its molecular identity complicates the identification of the many regulatory sites, which would help in drug design, allowing precise tuning of PTP opening.

## 3. Cyclophilin D: A Master PTP Regulator

To date, CyPD is one of the best-characterized protein interactors involved in the activation of the mammalian PTP without being a structural component and without affecting channel properties [33]. Initial studies to shed light on PTP modulation by CyPD involved protein inhibition or deletion, and overexpression of its encoding gene (*Ppif*). Clear-cut evidence in support of CyPD as a master regulator of the PTP came from the observation that *Ppif^-/-^* mice show a striking desensitization to Ca^2+^ of the PTP [34], which provides substantial protection from cell death in a number of PTP-related paradigms, including ischemia-reperfusion injury [34], heart failure [35], and muscular dystrophies [36,37]. How CyPD controls PTP opening is still a matter of debate. One hypothesis suggests a direct interaction with ANT [38,39], which could be enhanced under oxidative conditions [39]. CyPD can also bind F-ATP synthase in a process favored by Pi [40], providing a possible explanation for the modulation of the PTP by Pi.

Recent work pointed out that CyPD can undergo many different post-translational modifications (PTMs), including phosphorylation, oxidation, acetylation, and S-nitrosylation that control PTP opening, as recently reviewed in [41]. One of the most studied PTM is perhaps CyPD phosphorylation by glycogen synthase kinase 3β (GSK3β), a constitutively active Ser/Thr protein kinase that enhances PTP opening in cancer cells [42] and promotes CyPD binding to ANT [43]. A set of kinases known as RISK (reperfusion injury salvage kinases, including Akt, ERK1/2, PKG, PKC-ε, and p70s6K) performs an inactivating phosphorylation on GSK3β, impairing its ability to trigger the CyPD-PTP interaction [44]. Although the phosphorylation site(s) involved in the activity of GSK3β are yet unknown, the phosphorylation status at S191 or S42 directly impacts on the ability of CyPD to regulate the PTP. The phosphoresistant CyPD S191A mutation increases the resistance of the pore to Ca^2+^, protects against cell death and exhibits a reduced myocardium infarct size after ischemia [45]. In addition, phosphorylation at S42 increases the propensity for pore opening in mitochondria from MCU-KO mice [46]. It is interesting to note that the phosphorylation of both serine residues correlates with an enhanced CyPD binding to the F-ATP synthase. Other CyPD PTMs impinging on PTP modulation have been studied, such as sirtuin-3 (SIRT3) dependent deacetylation following SIRT3 overexpression [47,48,49]. In particular, deacetylation of CyPD at K166 inhibits PTP opening and reduces cell death [47].

In tumor cells, CyPD was found to be sequestered by TRAP1, the mitochondrial paralog of HSP90, which antagonizes the CyPD-dependent induction of PTP via its protein folding/unfolding mechanisms [50]. The formation of the TRAP1/CyPD complex is prevented by CyPD-targeting compounds such as cyclosporine A (CsA), but not by the TRAP1 ATPase activity inhibitor Gamitrinib, and can be disrupted by p53. TRAP1 has been directly connected to PTP modulation, given that its inhibition or downregulation results in mitochondrial depolarization, cytochrome c release, and cell death, which are all features of PTP activation, while its overexpression exerts a protective effect [51]. The TRAP1-dependent PTP regulation is detectable in different pathophysiological contexts, including models of neural stem cells, kidney disease, and ischemic damage [52,53,54]. Indeed, TRAP1 is overexpressed under hypoxia [55] and protects cardiomyocytes and rat brains from hypoxic injuries and ROS-dependent pore opening upon ischemia-reperfusion [56,57]. Whether the TRAP1-dependent PTP modulation occurs uniquely through CyPD sequestering is not known. Recently, TRAP1 was reported to directly bind several F-ATP synthase subunits, in particular β, α, γ, d, g, and OSCP subunits [58], and this may open a new field of investigation. A set of highly selective TRAP1 inhibitors were recently identified by applying a molecular dynamics-based approach [59]. These compounds (compound 5 in particular) inhibit TRAP1 ATPase activity abrogating its pro-tumorigenic functions without affecting HSP90. It will be important to understand whether this beneficial effect might also be due to an increased PTP opening propensity.

At present, PTP inhibition is achieved mostly by the use of drugs targeting CyPD (see Table 1). The best characterized CyPD-targeting PTP inhibitor is CsA [60,61,62]. The use of CsA in vivo, however, presents several intrinsic limits. One issue that is often not adequately considered is the expression level of CyPD, which may vary widely in different cells and tissues [63]. Additional problems arise from the inhibition of calcineurin. CsA indeed binds all cyclophilins, including the isoform A located in the cytosol forming the CsA-CyPA complex able to inhibit the Ca^2+^-dependent phosphatase calcineurin, preventing nuclear translocation of NFAT and consequent transcription of NFAT-targeted genes (e.g., IL2), resulting in immunosuppression [64,65,66]. Importantly, calcineurin inhibition also prevents dephosphorylation of Drp1 and its translocation to mitochondria, preventing fission through an effect that is unrelated to PTP inhibition [67]. To avoid these complex effects, derivatives of CsA such as NIM811 and Debio025 (also called Alisporivir) have been synthesized, which after binding cyclophilins cannot form complexes with calcineurin. These derivatives maintain the ability to inhibit PTP opening and have been successfully used in the treatment of several pathologies. Of note, these CsA derivatives showed a remarkable antiviral effect against HCV [68,69], and in the case of Alisporivir, also against HIV [70]. NIM811 improves skeletal muscle salvage and survival in vivo after ischemia-reperfusion injury [71] and ameliorates mitochondrial structural and functional abnormalities in several models of muscular dystrophies [72]. It also improves mitochondrial functions and decreases neurodegeneration after traumatic brain injury [73], and appears effective in protecting against acute pancreatitis in different models [74]. Another CyPD-targeting compound is sanglifehrin A (SfA), a macrolide produced by actinomycetes [75] with potent immunosuppressive features. SfA is not related to CsA, but like CsA, it tightly binds to CyPA, although the resulting complex does not inhibit calcineurin. Rather, SfA arrests T cell proliferation in the G1 phase in response to interleukin 2 through a mechanism involving NFkB-mediated upregulation of p53 and p21 genes, which inhibit cell cycle kinases. SfA acts as a potent PTP inhibitor by sequestering CyPD and was shown be protective against damage following ischemia-reperfusion [76]. However, all these compounds suffer from many disadvantages, including complex multistep synthesis, potential side effects unrelated to cyclophilin inhibition and cytotoxicity. Newly synthesized small CyPD inhibitors have been developed and applied in several pathological conditions. A fragment-based drug discovery approach was used to generate a new family of non-peptidic, small-molecule cyclophilin inhibitors with potent antiviral activity against HCV, human immunodeficiency virus, and coronaviruses [77]. In particular, compound 31 showed good efficacy in protecting mice from hepatic ischemia-reperfusion injury [78]. However, a general limitation of all these CyP-targeting compounds is that they are not PTP blockers and that their efficacy is limited by target availability.

## 4. Mitochondrial Permeability Pathways: The ANT and the F-ATP Synthase Hypotheses

The molecular identity of the PTP is a matter of a long-standing debate in the field. In spite of some controversies for which we refer to a recent review for a more detailed discussion [100], one of the current hypotheses proposes that both F-ATP synthase and the ANT can provide distinct IMM permeation pathways. Whether these two proteins represent distinct permeability pathways or whether they cooperate is still under investigation. The identification of regulatory mechanisms and compounds that act specifically on these proteins could provide important clues to dissect their specific involvement in pathophysiological conditions.

### 4.1. The ANT Pore

The ANT was the first candidate proposed for PTP formation as reviewed in [101,102]. It represents the most abundant protein of the IMM; in humans, four different isoforms exist and are encoded by genes with high sequence homology (~80–90%); in comparison, mice express only three ANT isoforms. In respiring mitochondria, it exchanges matrix ATP for cytosolic ADP (with a strict ratio 1:1) according to their concentration gradient and to the membrane potential, and generates a net negative charge. The transport mechanism consists of the alternation of two different conformations, i.e., the cytoplasmatic-state (c-state) and the matrix-state (m-state), which are opened toward the IMS and the matrix side, respectively. In both states, a complex network of electrostatic interactions closes the transporter from one side and allows the release of the nucleotide from the other side [103,104]. The carrier was indeed suggested to operate with a single binding center gated pore mechanism, by which the binding of ADP (or ATP) drives the conformational changes between the two states modifying the matrix and cytoplasmic salt bridge networks; in this way, the unique binding site is accessible for only one nucleotide at a time [90]. The structure of the ANT conformation states was revealed in the presence of specific inhibitors, ATR and bongkrekic acid (BKA), which freeze ANT in the c-state and m-state, respectively [104]. Supporting evidence for the ANT hypothesis as the PTP came from the ability of ATR and BKA to exert opposite effects on the PT, i.e., ATR promotes pore activation while BKA plays an inhibitory role. Another important clue concerns the channel activity of ANT. Reconstituted bovine ANT into proteoliposomes indeed gives rise to Ca^2+^-activated channels with subconductances of 300–600 pS showing, in addition, a clear voltage dependence that resembles that of the PTP [91]. ANT channel openings are also prevented by acidic pH, although at lower values (about 5.2) compared to those required for PTP inhibition (about 6.5) [91]. Furthermore, recombinant ANT derived from *Neurospora crassa* showed a similar profile of conductances, inhibition by ADP and BKA, and activation by CyPD, although this could not be completely prevented by CsA [92].

ANT was also proposed to mediate the effect of oxidants in PTP modulation. In particular, two ANT cysteines (C160 and C257) were found to be responsive to PAO and DIA generating direct cross-links and via glutathione, respectively, that would activate pore opening [105]. Moreover, the oxidation of an additional ANT cysteine residue (C56) was suggested to promote ANT dimerization that might facilitate channel formation [105]. However, a causal relationship between ANT cysteine modifications and pore modulation remains to be established, also considering that the ablation of ANT isoforms 1 and 2 did not prevent the effect of oxidants on the PTP, which still responded to DIA and TBH [27]. In spite of the fact that more than two decades have elapsed from this proposal, no clues have been provided to explain how ANT can form a channel. Considering the mechanism of adenine nucleotide transport, it appears that the internal cavity is sealed during the catalytic cycle, with the release or binding of the adenine nucleotide on opposite sides. This seal may be incomplete in the presence of free FAs, given that under these conditions, ANT can mediate H^+^ translocation across the inner membrane [106]. One cannot exclude that Ca^2+^ may promote a dramatic conformational change on ANT, which would then accommodate a high conductance channel, although this hypothesis requires experimental validation. The involvement of cardiolipin (which tightly binds ANT) in this putative Ca^2+^-dependent modification of ANT has also been suggested [107].

In summary, we think that the role of ANT in PTP formation is rather strong and can explain the modulation of the PT by both CyPD and ATR/BKA. However, whether ANT operates as a pore or whether it influences the opening of an alternative channel is still not known. It is important to note that the electrogenic ADP/ATP transport by ANT could itself affect the PT process by modulating mitochondrial surface potential [108], which may impinge on another permeability pathway. Genetic studies demonstrated that the PT cannot be entirely ascribed to ANT. In a recent work from the Molkentin laboratory, ANTs triple KO (TKO) mice lacking all murine isoforms (ANT1/2/4) have been characterized, revealing that the PT persisted although it had a higher Ca^2+^ threshold for activation [109]. Yet, TKO mitochondria underwent CyPD-dependent swelling in a sucrose-based medium, clearly indicating the existence of an ANT-independent permeability pathway. Consistently, TKO mitochondria still display channel activity as measured by patch-clamp, although at variance from wild-type, appears to be insensitive to ADP, suggesting that the inhibitory effect of ADP could be ascribed to ANT. These observations clearly demonstrate that the PT occurring in the absence of ANTs must be mediated by a distinct pathway, which could be provided by F-ATP synthase.

### 4.2. The F-ATP Synthase Pore

The F-ATP synthase is a multiprotein complex that primarily resides at the mitochondrial cristae edges. It consists of a globular, water-soluble F_1_ head (α3β3) and a membrane-embedded F_o_ subcomplex which includes subunit a and the c-ring. These two domains are linked by two stalks: the peripheral stalk (OSCP, b, d, F6 and in part A6L) and the central stalk (γ, δ, ε), which expands within the F_1_ head [110]. The peripheral stalk has a membrane domain that is structurally connected to the F_o_ and includes subunits e, g, f, part of A6L, 6.8PL, and DAPIT, which are involved in forming a complex interface between monomers [111]. This allows F-ATP synthase to organize into dimers or higher complexes, such as tetramers and oligomers, that contribute to determining the typical bending of cristae [112]. F-ATP synthase, and in particular the OSCP subunit of the peripheral stalk, was identified as a novel interactor of CyPD, which decreases the ATPase activity in a CsA-dependent manner [40,113]. Moreover, an ever-increasing number of studies provided robust evidence that F-ATP synthase is one of the best candidates for PTP formation. F-ATP synthase isolated from native gels from bovine [113], human [114], yeast [23], and drosophila [115] mitochondria, exhibited Ca^2+^-activated currents in planar lipid bilayers that can be inhibited by ADP/Mg^2+^. More recently, highly purified bovine F-ATP synthase extracted under very mild detergent conditions, i.e., in presence of lauryl maltose neopentyl glycol (LMNG), that preserves all subunits including the very labile DAPIT and 6.8PL, was shown to generate Ca^2+^-activated channels with features that perfectly match those of the PTP [82]. ADP/Mg^2+^ readily inhibited channel activity, indicating that the adenine nucleotide binding site within the catalytic domain of the F-ATP synthase is critical. In this study, dimers and oligomers gave rise to high conductance channels while monomers were inactive, strengthening the potential role of the dimerization interface in pore formation. Other groups pointed out the importance of monomers and proposed that a channel forms within the c-ring. Indeed, F-ATP synthase monomers extracted with dodecyl maltoside (DDM) generated channels that could be inhibited by ATP and Ba^2+^ and displayed conductances similar to those of the PTP [88]. Of note, these channels opened in the absence of added Ca^2+^, which only increased the frequency of events. The apparent discrepancy about the requirement of monomers or dimers may be explained by the difference in F-ATP synthase structure and/or subunit composition, which are determined by the specific detergent utilized. Indeed, treatment with DDM leads to the loss of subunits, e.g., DAPIT and 6.8PL; given its strong delipidating properties, this detergent may also alter the lipid plug within the c-ring [116] that may be critical for PTP formation. Thus, an intact peripheral stalk may be required for PTP activation through the c-ring in intact mitochondria.

Together with reconstitution studies, major efforts have been devoted to mapping critical PTP regulatory sites on the F-ATP synthase by subunit deletion and by site-directed mutagenesis. Mutation of a specific threonine residue (T165) in subunit β, which coordinates the binding of Mg^2+^ during catalysis, strongly lowers the sensitivity of the PTP to Ca^2+^, indicating that the binding site for the cation resides within the F_1_ domain [117]. OSCP is also a fundamental hub for PTP modulation. This subunit not only represents a binding site for CyPD, but also possesses a critical His (H135) that mediates the effect of pH on the PTP [118]. Furthermore, we recently reported that the unique OSCP Cys (C141), which is in proximity to the H135, is involved in the modulation of the PTP by oxidants [119]. In particular, this thiol which actively responds to DIA and mitoparaquat and modulates pore opening appears to be masked by the binding of CyPD, which exerts a protective role. The molecular consequence of C141 oxidation is still unclear, although we hypothesized that OSCP homodimers might form between F-ATP synthases of adjacent cristae, leading to a conformational change of the enzyme that could favor the transition toward pore formation. Site-directed mutagenesis in yeast did not provide compelling clues on the involvement of other conserved thiols (those located in subunits α, γ, and c) [119], although the participation of cysteines present uniquely in the mammalian enzyme cannot be ruled out. These findings suggest that other PTP-related proteins, such as ANT, might participate in the PTP modulation by oxidation together with the F-ATP synthase through its OSCP C141.

The site of channel formation within the F-ATP synthase is still a matter of debate. In support of the c-ring hypothesis, the substitution of key glycine residues of subunit c increased channel conductance of the c-ring [114] and accelerated calcein release in response to ionomycin [120]. Interestingly, a naturally occurring G87E variant of subunit c increased the PTP propensity to open, exacerbating mitochondrial damage in patients with ST elevation myocardial infarction (STEMI) [121]. In support of the dimer/tetramer hypothesis, ablation of subunits e and g in yeast desensitized PTP to Ca^2+^, decreased swelling, and reduced F-ATP synthase channel conductance up to tenfold [23,122,123]. Consistent with the role of dimers in channel formation, single substitutions of R8 of subunit e and E83 of subunit g (which entail electrostatic interactions stabilizing the dimeric complex) gave rise to smaller channels that never reached the full conductance state [124]. Thus, subunits e and g might represent a strict requirement for the formation of a full conductance PTP channel, although other subunits can be involved. For instance, the lack of subunit f, which is another small constituent of the Fo domain in close contact with subunits e and g, affected the PTP Ca^2+^-dependence and swelling capacity [125]. Altogether these findings strongly support the primary contribution of F-ATP synthase to PTP formation, yet the molecular mechanism that switches this energy-conserving enzyme to an energy-dissipating system remains to be defined. Recent cryo-EM studies showed peculiar F-ATP synthase structures obtained in the presence of Ca^2+^, which were never observed with Mg^2+^ that could potentially describe different conformational states in the process of PTP opening [116].

### 4.3. F-ATP Synthase and ANT Mediate Distinct Permeability Pathways

Evidence against the involvement of F-ATP synthase in the PT process derives from a set of thorough experiments in cells with genetically modified F-ATP synthase. It was shown that a Ca^2+^ and CsA-sensitive PT occurred in HAP1 cells deleted of subunits c [126], OSCP [127], b [127], or c and δ [128]. Since these deletions prevented the assembly of the F-ATP synthase, it was concluded that the enzyme is not involved in the formation of the PTP. However, Neginskaya et al. [129] measured the electrophysiological properties of HAP1-A12 mitoplasts (devoid of subunit c) and found that, differently from parental mitoplasts, they did not show high-amplitude currents in response to Ca^2+^, but rather smaller conductances (about 300 pS) which strongly resembled those of reconstituted ANT [92]. Consistent with a potential role of ANT in the generation of these currents, BKA was shown to force the transition to smaller conductances which could then be completely inhibited by ADP and CsA. In good agreement with an ANT-mediated PT, HAP1-A12 cells underwent Ca^2+^-dependent depolarization, which could be prevented by BKA that did not cause inhibition in parental cells. These findings pointed out that in the absence of a fully assembled F-ATP synthase, the PT could be mediated by ANT forming a distinct permeability pathway. In support of this hypothesis, Carrer et al. provided further insights by analyzing the electrophysiological properties of mitoplasts isolated from HAP1 cells lacking subunit b or OSCP and from HeLa cells genetically ablated for subunit g [130]. In wild-type cells and mitoplasts, PTP opening could be efficiently inhibited by CsA, but was completely refractory to BKA, suggesting that the ANT pore (also referred to as A-PTP) did not emerge in the first place, while the F-ATP synthase pore (also referred to as F-PTP) appears to predominate. The lack of subunit b or OSCP, which generates vestigial F-ATP synthases [127] and likely prevents channel formation by the enzyme, unmasks the A-PTP, as confirmed by the efficacy of BKA in blocking channel activity in situ and the PT in living cells [130]. The ablation of subunit g, which also caused the loss of subunit e, completely prevented PTP opening unless ATR was added and forced A-PTP activation. Different from mitoplasts devoid of subunit c, which showed channels sensitive to both BKA and CsA [129], mitoplasts with a defective F-ATP synthase peripheral stalk (i.e., absence of OSCP or b subunits) became sensitive to BKA but were refractory to CsA [130]. This puzzling observation could help to shed light on a potential structural relationship between ANT and the F-ATP synthase, which were shown to physically interact in the so-called “ATP synthasome” and respond in a still undefined manner to CyPD [131,132].

## 5. Pharmacology of the PTP: Hunting for the Target

The possibility to target the PTP is of particular interest considering its implications in the physiological homeostasis of both Ca^2+^ [133,134,135,136,137] and ROS [138] as well as in the activation of the cell death cascade [139]. PTP openings in the “long-lasting, high-conductance” mode, often followed by mitochondrial matrix swelling, rupture of the outer membrane, and release of proapoptotic factors, are associated with cell death initiation and have been extensively associated with several pathological conditions. As already mentioned, through the use of CsA or of its non-immunosuppressive derivatives or through the genetic ablation of *Ppif*, key advances have been made in understanding the implication of PTP in disease, and to date, the number of PTP-related paradigms has significantly increased. Among these, ischemia-reperfusion injury, muscular dystrophies and neurological disorders are perhaps the best characterized. The PTP indeed represents a promising target for cardioprotection under ischemia-reperfusion [140]. In the heart, mitochondrial dysfunction accelerates when blood flow is re-established after prolonged ischemia; while ischemic condition does not cause PTP opening itself, likely because of the protective effects of intracellular acidosis [141], it facilitates PTP opening at the reperfusion phase. Evidence that the PTP plays a role in reperfusion injury has been obtained in several experimental models, including isolated cardiomyocytes [142], perfused hearts [143], and in living animals [34,35]. PTP activation has also been linked to excitotoxicity, which is accompanied by a massive Ca^2+^ influx and a consequent neuronal cell death [144,145] and to mitochondrial alterations in Reye’s syndrome [146], in multiple sclerosis [147], amyotrophic lateral sclerosis [148] and Alzheimer’s disease [149,150]. In the latter, amyloid beta, which forms aggregates in the patient’s brain were shown to bind CyPD, and this correlates with an enhanced susceptibility of PTP opening [149]. PTP-dependent mitochondrial dysfunction has been demonstrated to play a pivotal role in muscular dystrophies [151]. Collagen VI-deficient mice which recapitulate muscle defects observed in Bethlem patients, could be recovered by treatment with CsA or with Debio025 [152] or by crossing with *Ppif^–/–^* mice [37]. Remarkably, these findings have also been extended to various genetic models of Duchenne muscular dystrophy [36,79,80].

However, emerging evidence points to potentially deleterious consequences of deregulations of PTP openings also in the “low-conductance” mode, which mostly impacts on Ca^2+^ and ROS homeostasis. Indeed, decreased PTP flickering due to a decreased level of CyPD acetylation has been recently causally associated with the onset of spastic paraplegia, and the restoration of a physiological PTP activity can rescue neuronal function both in vitro and in vivo [153].

**Figure 1:**
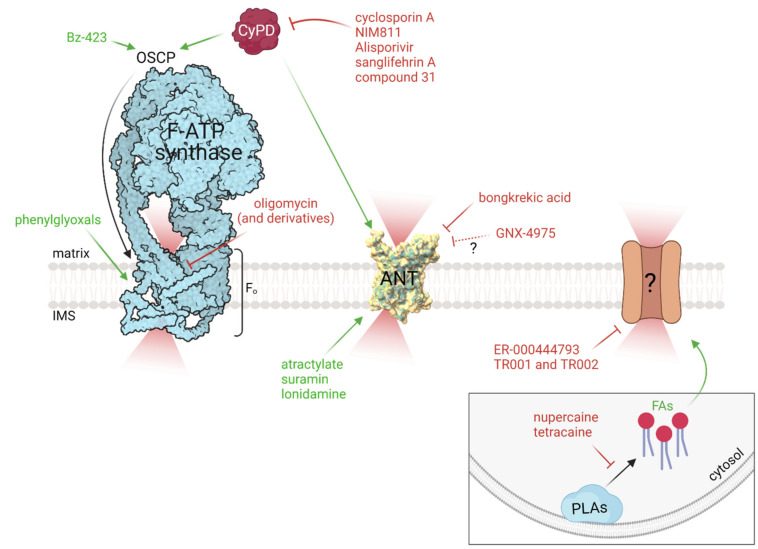
Exogenous modulators of the permeability transition (PT) and related targets. Schematic representation of exogenous PT modulators impinging on F-ATP synthase, adenine nucleotide translocator (ANT), cyclophilin D (CyPD), or still unknown target(s). Activators and inhibitors are marked in green and red, respectively. Pore formation within the Fo domain of the F-ATP synthase might be triggered by a conformational change (marked with a black arrow) favored by CyPD and/or Benzodiazepine-423 (Bz-423) binding to OSCP subunit, or phenylglyoxals, while it could be potentially inhibited by oligomycin (and derivatives). Compounds that sequester CyPD (cyclosporin A, NIM811, Alisporivir, sanglifehrin A, and compound 31) prevent its binding to F-ATP synthase and/or to ANT inhibit the PT process. Pore formation by ANT is favored by atractylate, suramin, or lonidamine, while it is prevented by bongkrekic acid and potentially by cinnamic anilides (such as GNX-4975). Nupercaine and tetracaine inhibit phospholipases (PLAs) and prevent the release of fatty acids (FAs), which favor PT occurrence through a not yet defined mechanism. The target(s) of PT inhibitors like ER-000444793 and triazoles (such as TR001 and TR002) remain(s) to be defined.

Lack of a full understanding of the PTP constituents certainly represents a complication for any PTP-targeting strategy, also in light of the fact that CyPD could not always be involved in PTP modulation. Although recent advances in the identification of the PTP molecular identity have been made, the still uncertain molecular mechanism for pore formation by the two leading candidates makes standard target-based approaches problematic. Both F-ATP synthase and ANT indeed play a pivotal role in the energy-conserving circuit and they cannot be easily targeted without altering the bioenergetic balance. In the next section, we will discuss a set of compounds (Table 1) that impinges on F-ATP synthase and on ANT as well other molecules with unknown targets that modulate the PT (Figure 1). We would like to stress that some compounds discussed below are not suitable for pharmacological purposes because of the ability to interfere with ATP synthesis or adenine nucleotide transport, yet they could represent a useful tool to shed light on the involvement of the two permeability pathways under pathological conditions.

### 5.1. F-ATP Synthase-Targeting Compounds

#### 5.1.1. Benzodiazepine-423

Benzodiazepine (Bz)-423 causes selective cytotoxicity in lymphocytes involved in autoimmune disorders [81]. For this reason, Bz-423 was proposed as a lead drug to treat systemic lupus erythematosus. Concerning its mechanism of action, the initial hypothesis was that Bz-423 initiates the apoptotic process through the generation of superoxide anion and Bax/Bak dependent cytochrome c release [154]. Bz-423 was then shown to target the F-ATP synthase at the OSCP subunit, resulting in a significant decrease of enzymatic activity, mimicking the action of CyPD on the F-ATP synthase [155]. Bz-423 and CyPD compete for binding OSCP, and both lower the Ca^2+^ threshold required for PTP opening [113]. Bz-423 can be seen as a small molecule “analog” of CyPD for the effects on the PTP, as also indicated by its promoting effect on channel activity of reconstituted F-ATP synthase [82]. Taken together, these findings strongly support that the effect of Bz-423 on the PT is mediated by its interaction with the F-ATP synthase.

#### 5.1.2. Phenylglyoxals

Phenylglyoxals (PGO) specifically target arginine residues, with which they form stable derivatives through their guanidino group, particularly under mildly alkaline conditions [83]. Rat liver mitochondria pretreated with PGO are more resistant to PT occurrence triggered by an uncoupler, suggesting that (i) PGO adducts affect the open probability of the PTP, and (ii) critical arginine residues are involved in PTP modulation [25,26,83,84,85]. This modulatory mechanism appears to be species-specific. While PGO desensitizes PTP opening in rat, mouse, and yeast mitochondria, it sensitizes the PTP to Ca^2+^ in drosophila and human mitochondria [86]. In yeast, the critical arginine residue targeted by PGO and mediating the effect of PGO on the PT has been identified in position 107 of F-ATP synthase subunit g. Remarkably, the expression of human subunit g in yeast shifts the effect of PGO on the PTP from inhibition to activation, proving that the species specificity of PTP modulation by PGO relies on the R107 subunit g [86]. In keeping with a specific effect on F-ATP synthase, PGO was shown to negatively modulate the enzymatic activity; the effect is more pronounced when Ca^2+^ (but not Mg^2+^) was used [156]. PGO derivatives that differ for the functional groups attached to the phenyl ring lead to different consequences on PT [83]. At variance from PGO, OH-PGO triggers the permeability transition in rat mitochondria, suggesting that the overall charge of the adduct influences PTP voltage sensing.

#### 5.1.3. Oligomycin and Related Compounds

Among F-ATP synthase inhibitors, oligomycin is perhaps the best characterized. The binding site is located in between two adjacent c-subunits in contact with the proton half-channel formed by subunit a, with a subsequent block of proton transport [157]. Thus, the number of bound oligomycin molecules for one F-ATP synthase was supposed to be limited to the number of c helixes exposed to the proton half-channel, yet at least seven molecules were modeled in the crystal structure, suggesting that more binding sites are possible [157]. While the inhibitory mechanism of oligomycin on the enzymatic activity of the F-ATP synthase is well documented, the consequences of oligomycin treatment are more complex than expected. Indeed, after the initial inhibition of ATP synthesis (which causes a drop of mitochondrial respiration linked to ADP phosphorylation), oligomycin can stimulate oxygen consumption [158]. This phenomenon was explained by the unexpected uncoupling activity of oligomycin, which appears to be inhibited by ATP, given that both glycolysis-supplied ATP or maintenance of matrix ATP by BKA abolish this uncoupling activity [158]. Of note, CsA does not protect against oligomycin-mediated uncoupling, indicating that the PTP is not involved in such a mechanism. Oligomycin is indeed not considered to be a PTP modulator. A number of studies aimed at defining PTP regulatory mechanisms included oligomycin in the experimental protocol in order to preserve the ATP/ADP balance, which could in turn indirectly affect the propensity for PTP opening [13,24,87]. However, several reports described the potential of oligomycin as a PTP inhibitor under particular conditions. Oligomycin prevents PT initiated by Bax [159,160] or by a high concentration of selenite [161] and differently from CsA, inhibition by oligomycin could be released by FCCP addition [162]. In another set of experiments, the inhibitory effect of oligomycin appeared to be additive to that of CsA [163] and to require Pi, given that the replacement of this anion with acetate abolished oligomycin-mediated PTP inhibition [162,163,164]. Oligomycin can also prevent the PT induced by ATR, likely by increasing the ADP/ATP ratio, which in turn may hinder the effect of ATR [165,166].

Whether oligomycin exerts this dampening on the PTP by binding the F-ATP synthase is not known. In this regard, oligomycin as well as other F-ATP synthase inhibitors (e.g., venturicidin) that bind to a unique pocket within the c-ring, were reported to decrease calcein release upon stimulation with a Ca^2+^ ionophore [120]. More strikingly, channel activity of DDM-solubilized F-ATP synthase monomers showed a clear-cut inhibition by oligomycin [88], pointing to a possible direct effect on the PTP-forming ability of the enzyme. As already mentioned, DDM, which efficiently dissociates hydrophobic protein-protein interactions and alters subunit composition of F-ATP synthase [167], may cause conformational changes on the enzyme and/or unmask binding sites for oligomycin that are not accessible in the absence of detergent. Recently, a first target-based approach considering the c-ring as a primary candidate for PTP formation has been carried out. Oligomycin-based small molecules have been developed aiming at discovering new potential PTP modulators [89]. These compounds (such as compound 10) based on the 1,3,8-triazaspiro[4,5]decane scaffold specifically targeted the c subunit and showed good PTP inhibitory capacity without providing apparent off-target effects or alterations in mitochondrial bioenergetics. Interestingly, these compounds also displayed beneficial effects in preventing cell death during the reperfusion phase in a model of myocardial infarction [89].

### 5.2. ANT-Targeting Compounds

As already mentioned, the early literature on the PT reported the effect of the ANT ligands, ATR and BKA, in stimulating or antagonizing pore opening, respectively [2]. ATR is a diterpenoid glycoside of 803 kDa extracted from *Atractylis gummifera* and other plants, the toxicity of which was widely documented in the last two centuries [168]. The major biological effects of ATR and of its derivatives, like carboxyatractyloside, depend on the inhibition of the ANT translocase activity, which arrests oxidative phosphorylation [169]. ATR, locking ANT in the c-state, prevents ADP binding to the transporter [170]. ADP that has a highly similar geometry and charge distribution as ATR, exerting a strong competition for the same binding site on the ANT. BKA is an unsaturated tricarboxylic fatty acid of 486 kDa produced by the bacterium *Burkholderia gladioli pathovar cocovenans* [171], which freezes ANT in the m-state preventing the ADP/ATP exchange [172]. The mechanism of action of these compounds in modulating the PT has not been resolved. Together with a conformational change on the ANT, one possibility is that ATR might act by preventing the binding of adenine nucleotides to the external carrier site, whereas BKA prevents the dissociation of the adenine nucleotides from the internal site [173]. In addition to ATR and BKA, several molecules have been found to efficiently modulate ANT translocase activity [174]. Suramin, which efficiently inhibits ANT activity, also triggers mitochondrial swelling in an ADP- and CsA-sensitive fashion, possibly by oxidizing critical thiols [93]. Interestingly, suramin is a drug already approved by the FDA with multiple pharmacological activities, including inhibition of P2Y2 receptors [94]. Lonidamine is a derivative of indazole-3-carboxylic acid and is known to inhibit aerobic glycolysis and energy metabolism selectively in tumor cells. Lonidamine was also shown to trigger PTP opening in a CsA-sensitive manner [175]. Interestingly, lonidamine elicited ANT channel activity in planar lipid bilayers [95], suggesting that it exerts a direct effect on channel formation by the ANT.

### 5.3. CyPD-Independent PTP Inhibitors

A first high throughput screening (HTS) of commercially available small molecule libraries led to the discovery of at least four classes of new low molecular weight PTP inhibitors. The first class included cinnamic anilide derivatives, which were able to efficiently inhibit mitochondrial swelling triggered by various stimuli (Ca^2+^ and oxidants) and to improve Ca^2+^ retention capacity (CRC) more efficiently than CsA [176]. One such compound (GNX-4728) demonstrated protective effects against ischemia-reperfusion injury and in a mouse model of amyotrophic lateral sclerosis, where it improved motor function and limited neurodegeneration [96], while it lacked therapeutic efficacy in mouse neonatal hypoxia-ischemia [177]. The mechanism of action of these compounds is still not known. One hypothesis has been advanced by Halestrap and co-workers, who showed that that the number of binding sites for another cinnamic anilide derivative (GNX-4975) depends on the conformation assumed by the PTP. They also proposed that GNX-4975 enhances the association of ANT to the phosphate carrier and potentially prevents a Ca^2+^-triggered-conformational change that would open the ANT interface into a pore [178]. However, this hypothesis still requires direct experimental validation.

ER-000444793 is another small CyPD-independent inhibitor derived from an HTS of 50,000 compounds that decreased mitochondrial swelling while increasing Ca^2+^ retention capacity in a dose-dependent manner [99]. ER-000444793 failed to affect ATP synthesis up to a concentration of 50 μM, suggesting, reassuringly, the absence of effects on the catalytic activity of the complex, although a potential effect on F-ATP synthase organization and stability remains to be addressed.

Another HTS of the NIH Molecular Libraries Small Molecule Repository collection of 363,827 compounds, followed by optimization, used mitochondrial swelling, CRC and maintenance of membrane potential to identify two classes of PTP inhibitors with picomolar inhibitory activity, i.e., isoxazoles [179] and benzamides [180]. Both classes of compounds displayed synergistic effects with CsA and did not affect ATP synthesis or mitochondrial respiration. From isoxazoles, second-generation compounds possessing a triazole in place of the core isoxazole have been developed, showing improved plasma stability [181]. TR001 and TR002 are amongst the most potent PTP inhibitors, which have been successfully applied in vivo to improve survival in a zebrafish muscular dystrophy model [97] and decrease ischemia-reperfusion damage in perfused mouse hearts [98], respectively.

### 5.4. Fatty Acids and Phospholipase Inhibitors

The mechanism(s) through which FAs stimulate pore opening is an open issue [182]. Alterations of mitochondrial membrane stability by lipid extracts resulting in uncoupling were known since the early 1950s. This uncoupling factor [29,183] was identified as a mixture of long-chain non-esterified fatty acids with a more potent activity showed by unsaturated fatty acids, e.g., oleic, linoleic, linolenic, and arachidonic acid [184]. The FAs-dependent enhancement of membrane leakage was initially attributed to their protonophoric effect, but this process appears to occur over minutes (at least for long-chain fatty acids), a time-frame that cannot account for the observed kinetics of dissipation of the proton motive force [185]. One hypothesis was that FAs do not act as protonophores as such, but rather require an additional mitochondrial component, which was then identified to be the ANT [186,187]. Data in support of this were that (i) ANT inhibitors abolished the FAs-mediated uncoupling [188,189], and (ii) reconstituted ANT showed proton channel activity in response to FAs [190]. The addition of FAs to mitochondrial preparations not only uncoupled oxidative phosphorylation but also led to large-amplitude swelling, thus activating a permeability transition process [183]. Interestingly, the mitochondrial swelling capacity upon the addition of FAs strictly depends on the chain length of the FA itself and on the presence of double bonds, which is consistent with the protonophoric activity. The role of FAs in promoting PTP opening and cell death was documented by Penzo et al. [191], who also showed that the minimal number of unsaturations required increases along with the chain length. Whether FAs lead to pore opening because of their protonophoric activity or because they can directly target PTP constituents is still not completely understood. FAs could modulate PTP opening by affecting membrane surface potential, which could be detected by voltage-sensing elements [192] or through a possible interaction with the pore complex [182]. For example, arachidonic acid was shown to positively modulate PTP opening accompanied by cytochrome c release independently of its uncoupling capacity [193].

One of the first demonstrations that FAs are involved in mitochondrial damage comes from the evidence that nupercaine, a well-known inhibitor of PLAs, maintained mitochondria tightly coupled for long periods of time, preventing in vitro aging. Mitochondrial damage, in this case, was attributed to a gradual degradation of mitochondrial phospholipids during storage, which could be inhibited by phospholipase inhibitor nupercaine [194]. Nupercaine was then used to prevent Ca^2+^-dependent swelling of NEM-treated mitochondria, a condition under which very low amounts of exogenous FAs can profoundly stimulate the rate of swelling [1]. Of note, tetracaine, another PLAs inhibitor, was shown to inhibit PTP opening in drosophila mitochondria [20]. The effect was immediate, which suggests a direct interaction with pore components rather than inhibition of PLAs.

## 6. Conclusions

Over the last few years, substantial progress has been made in the identification of the molecular nature of the PT. Channel formation by both F-ATP synthase and ANT has provided a very useful framework that allows to solve many apparent discrepancies in the literature and to accommodate a variety of effectors. Defining the detailed mechanisms of channel formation remains a challenge, but one that can now be addressed by mutagenesis and structure determination with high-resolution techniques. With these approaches, it will also be possible to readdress the role of the PT in pathophysiology.

## Figures and Tables

**Table 1:** List of compounds that modulate PT occurrence. When feasible, the target(s) and the mechanism of action are reported.

Compound Name	Target	Effect on the Permeability Transition (PT)	Mechanism of Action	Comments
cyclosporin A (CsA)	cyclophilins	inhibition	CyPD sequestration	CsA/CyPA complex inhibits calcineurin [64,65,66]
NIM811				effective in ischemia/reperfusion injury, dystrophic models, traumatic brain injury and pancreatitis [71,72,73,74]
Debio025 (or Alisporivir)				effective in dystrophic models [36,79,80]
sanglifehrin A (SfA)				effective in ischemia-reperfusion injury [76]
compound 31				effective in hepatic ischemia/reperfusion injury [78]
Benzodiazepine-423 (Bz-423)	F-ATP synthase (OSCP subunit)	activation	possible conformational change of the F-ATP synthase	induction of cell death in lymphocytes [81]; effective on channel activity of reconstituted F-ATP synthase [82]
phenylglyoxals (PGO)	F-ATP synthase (subunit g)	activation or inhibition (species-specific)	arginine adducts	[25,26,83,84,85,86]
oligomycin	F-ATP synthase (subunit c and a)	inhibition (controversial)	ND	no alterations of PT occurrence [13,24,87]; inhibition of channel activity of DDM-extracted F-ATP synthase [88]
compound 10	F-ATP synthase (subunit c)	inhibition	ND	protection in an ex vivo model of myocardial infarction [89]
atractylate (ATR)	adenine nucleotide translocator (ANT)	activation	blockage of ANT in the c-state	[90,91,92]
bongkrekic acid (BKA)		inhibition	blockage of ANT in the m-state	[90,91,92]
suramin		activation	oxidation of critical thiols	induction of mitochondrial swelling [93]; FDA approved [94]
lonidamine		activation	ND	activation of ANT channel activity [95]
GNX-4728 and GNX-4975 (cinnamic anilides)	adenine nucleotide translocator (ANT)?	inhibition	stabilization of ANT and phosphate carrier (PiC) interaction	effective in amyotrophic lateral sclerosis [96]
TR001 and TR002	ND		ND	effective in dystrophic models and in ischemia/reperfusion injury [97,98]
nupercaine and tetracaine	phospholipases (PLAs)		prevention of fatty acid release	[1,20]
ER-000444793	ND		ND	[99]
